# Analysis of factors associated with cognitive impairment in elderly patients hospitalized with chronic heart failure

**DOI:** 10.3389/fmed.2026.1799776

**Published:** 2026-05-25

**Authors:** Xiao-ying Jin, Han Li, Meng-dan Chu, Kai-xin Peng, Bo-wen Chang, Ke-han Hu, Zeng-feng Su

**Affiliations:** Department of General Medicine, The Fourth Affiliated Hospital of Anhui Medical University (Affiliated Chaohu Hospital), Chaohu, China

**Keywords:** chronic heart failure, cognitive impairment, elderly patients, associated factors, Mini-Mental State Examination

## Abstract

**Objective:**

This study systematically investigates the prevalence of cognitive impairment (CI) and its associated factors among older inpatients with chronic heart failure (CHF), aiming to inform early clinical screening and targeted interventions.

**Methods:**

A cross-sectional study consecutively enrolled 220 CHF inpatients aged ≥ 60 years from the Department of Cardiology and General Medicine at the Fourth Affiliated Hospital of Anhui Medical University between October 2024 and November 2025. Cognitive function was assessed using the Chinese version of the Mini-Mental State Examination (MMSE). Demographic, clinical, and laboratory data–including NT-proBNP, lipid profiles, and left ventricular ejection fraction (LVEF) –were collected. Intergroup comparisons were performed, and variables significant in univariate analysis (*P* < 0.05) were entered into multivariate logistic regression to identify independent predictors. Random forest variable importance analysis was conducted to validate model stability. Results: the prevalence of CI was 55.9%. Univariate analysis revealed significant differences in age, education, LVEF, smoking history, diabetes, and sleep quality (all *P* < 0.05). Multivariate logistic regression identified age ≥ 80 years (OR = 6.122, 95% CI: 2.695–27.979, *P* < 0.001), sleep disturbance (OR = 2.870, *P* = 0.016), diabetes (OR = 2.409, *P* = 0.040), and elevated NT-proBNP (>1000 pg/mL) as independent risk factors for CI. In contrast, education level of senior high school or above (OR = 0.186, *P* = 0.007) and LVEF ≥ 50% (OR = 0.035, *P* < 0.001) were protective. Random forest analysis confirmed LVEF as the most influential variable, followed by age and NT-proBNP.

**Conclusion:**

Cognitive impairment is highly prevalent among older inpatients with CHF and is influenced by advanced age, elevated NT-proBNP, sleep disorders, diabetes, lower education, and reduced LVEF. These findings highlight the high prevalence and associated factors of cognitive impairment in elderly patients with chronic heart failure, which may enhance clinical awareness of cognitive status in this population.

## 1 Introduction

Heart failure (HF) represents the terminal stage of various cardiovascular diseases, with its incidence rising markedly with advancing age. Approximately 26 million individuals are affected globally, and the prevalence among those aged over 70 years reaches up to 10% (1). In Asian populations, the overall prevalence of HF ranges from 1.3% to 6.7% (2), with a Chinese survey conducted between 2012 and 2015 reporting a rate of 1.3% (3). HF not only impairs cardiac structure and function but also compromises the physiological activities of multiple organ systems. Cognitive impairment (CI) refers to deficits in learning, memory, and executive judgment resulting from abnormalities in higher-order cerebral processing, encompassing both mild cognitive impairment (MCI) and dementia (4). MCI is a transitional state between normal aging and dementia, with a prevalence of approximately 6% among individuals aged 60–64 years and up to 25% among those aged 80–84 years (5). Both chronic heart failure (CHF) and CI are prevalent conditions in older adults, each contributing to elevated mortality, diminished quality of life, and substantial healthcare burdens. Evidence supports an independent association between CHF and CI: a 15-years cohort study involving 13,160 participants revealed that the proportion of CHF patients progressing to MCI or dementia (44.9%) was significantly higher than that of controls (34.4%) (6). The reported prevalence of CI among Asian patients with HF is high (44%) (7), however, data specific to the Chinese population remain limited. CI further worsens outcomes in HF patients, increasing the risk of mortality (HR = 1.33, 95% CI: 1.02–1.73) and rehospitalization (8), and impairs quality of life through diminished self-care capacity (9). The pathogenesis of post-HF cognitive impairment involves multiple factors, including reduced cerebral blood flow, structural brain alterations (10), decreased left ventricular ejection fraction, and comorbidities such as diabetes and atrial fibrillation (11). Nonetheless, definitive strategies for ameliorating cognitive function in HF patients are currently lacking.

This study aims to investigate the factors associated with CI among older inpatients with CHF, to clarify the effect sizes of potential risk factors, and to provide an evidence-based foundation for the early identification of high-risk populations and the formulation of targeted preventive and management strategies in clinical practice.

## 2 Study subjects and sample size

### 2.1 Research subjects

This study was a single-center, cross-sectional investigation. A total of 220 consecutive patients aged ≥ 60 years with chronic heart failure (CHF) who were hospitalized in the Department of Cardiology and the Department of General Medicine at the Fourth Affiliated Hospital of Anhui Medical University between October 2024 and November 2025 were enrolled based on predefined inclusion and exclusion criteria. The cohort comprised 111 males and 109 females. Inclusion criteria: eligible participants met the following criteria: (1) diagnosis of CHF in accordance with the 2024 Chinese Guidelines for the Diagnosis and Treatment of Heart Failure; (2) age ≥ 60 years; (3) New York Heart Association (NYHA) functional class ≥ II; (4) availability of complete clinical data; and (5) provision of written informed consent by the patient or their legal representative. Exclusion criteria: patients were excluded if they met any of the following conditions: (1) history of traumatic brain injury or acute cerebrovascular disease; history of old cerebral infarction was collected as a baseline variable; (2) pre-existing auditory or visual impairment that would preclude cognitive assessment; (3) concomitant malignant tumors or severe functional impairment of vital organs, including the liver, lungs, or kidneys; (4) cognitive decline attributable to causes other than CHF; (5) history of alcohol or substance dependence; or (6) incomplete clinical records. Ethical Approval: this study was reviewed and approved by the Ethics Committee of the Fourth Affiliated Hospital of Anhui Medical University (Approval No.: KYXM-202405-040). Verbal informed consent was obtained from all participants after full explanation of the study purpose and procedures, in accordance with the approval of the Ethics Committee.

### 2.2 Sample size determination

This cross-sectional study aimed to identify independent factors associated with cognitive impairment (CI) in older inpatients with chronic heart failure (CHF) using binary logistic regression. Sample size estimation was guided by the events per variable (EPV) principle proposed by Peduzzi et al., (12) which recommends a minimum of 10–15 positive outcome events per predictor variable to ensure model stability and avoid overfitting. Based on a planned inclusion of eight candidate predictors encompassing demographic characteristics, clinical comorbidities, and heart failure–specific indices, a minimum of 80 CI-positive events was required (8 × 10). Given a reported CI prevalence of 31%–85% in older CHF inpatients and a projected positive rate of 50% derived from pilot data (13), the estimated base sample size was 160 participants (80 ÷ 0.5). After adjusting for an anticipated 5% rate of incomplete data or invalid assessments, a minimum target sample of 168 participants was established. A total of 241 eligible older CHF inpatients were screened between October 2024 and November 2025. Following rigorous data collection and cognitive assessment, 220 participants with complete and valid records were included in the final analysis, among whom 123 were classified as CI-positive. Ultimately, seven candidate predictors were entered into the multivariate logistic regression model, with six retained as significant correlates, yielding an EPV ratio of 17.6:1 (123/7). This ratio substantially exceeded the recommended threshold, ensuring the robustness, reliability, and statistical validity of the identified associations.

## 3 Materials and methods

### 3.1 Collection of clinical data and assessment of cognitive function

Comprehensive clinical data were collected for all enrolled participants. Demographic characteristics included sex, age, height, weight, marital status, and educational attainment. Lifestyle factors comprised smoking history, alcohol consumption, and self-reported sleep quality. Medical history was documented for prior myocardial infarction, diabetes mellitus, hypertension, and stroke. Heart failure–specific clinical data included HF etiology and disease duration. Based on left ventricular ejection fraction (LVEF) measured by transthoracic echocardiography, patients were classified into three categories in accordance with current guidelines: heart failure with reduced ejection fraction (HFrEF, LVEF ≤ 40%), heart failure with mildly reduced ejection fraction (HFmrEF, LVEF 41%–49%), and heart failure with preserved ejection fraction (HFpEF, LVEF ≥ 50%). For patients classified as HFpEF, the diagnosis was further confirmed by the presence of objective evidence of structural heart disease, diastolic dysfunction, or elevated filling pressures (e.g., elevated natriuretic peptides and left atrial enlargement), as defined by the ESC 2021/HFA-PEFF diagnostic algorithm, to ensure diagnostic specificity beyond a normal ejection fraction alone. Laboratory parameters measured included high-density lipoprotein cholesterol, low-density lipoprotein cholesterol, triglycerides, homocysteine, uric acid, and N-terminal pro-B-type natriuretic peptide (NT-proBNP). Consistent with the 2024 Chinese Guidelines for the Diagnosis and Treatment of Heart Failure and established international cohort studies (PRIDE/ICON) (14). Data collection and cognitive assessment were performed after hemodynamic stabilization during hospitalization. Cognitive function was assessed using the Chinese version of the Mini-Mental State Examination (MMSE) by trained research personnel following standardized instructions. All assessments were administered face-to-face and verified on-site to ensure data authenticity and completeness. Cognitive impairment was defined according to established, education-adjusted MMSE cutoff scores: ≤17 for participants with no formal education, ≤20 for those with primary school education, and ≤24 for those with middle school education or higher (15). These thresholds are widely adopted in Chinese population-based studies to account for literacy-related performance variations. Written informed consent was obtained from all participants or their legally authorized representatives prior to enrollment.

### 3.2 Definition and grouping criteria of independent variables

The definitions and grouping criteria of each observation indicator in this study are as follows: a brief sleep assessment was used in this study. Sleep duration of 6–8 h per night was defined as good sleep, while sleep duration < 6 h, easy awakening, or difficulty falling asleep was classified as poor sleep. Standardized scales such as the PSQI may be adopted in future studies to further evaluate sleep quality. Smoking exposure was evaluated based on pack-years (cigarettes per day × years of smoking). Non-smoking was defined as “no,” and ≤100 pack-years, 100–200 pack-years, and >200 pack-years were combined as “yes,” which was finally converted into a binary variable. Diabetes mellitus was defined according to clinical diagnostic criteria, including fasting blood glucose ≥ 7.0 mmol/L, 2-h postprandial blood glucose ≥ 11.1 mmol/L, or a previous clinical diagnosis of diabetes with regular hypoglycemic treatment.

### 3.3 Quality control

Data collection and questionnaire surveys are conducted by investigators who have received standardized training. The relevant scale content is explained in detail to the respondents to ensure they understand it. The questionnaires are completed in a one-question-one-answer format, and if any issues arise during the process, the investigators provide standardized explanations to ensure data accuracy.

### 3.4 Statistical analysis

Normally distributed continuous variables are expressed as mean ± standard deviation (SD) and compared between groups using the independent samples *t*-test. Non-normally distributed continuous variables are presented as median (25th–75th percentile) and analyzed using the Mann–Whitney U test. Categorical variables are reported as frequencies (n) and percentages (%) and compared using the chi-square (χ^2^) test or Fisher’s exact test when expected cell frequencies were <5. Variables demonstrating statistical significance (*P* < 0.05) in univariate analyses, along with those deemed clinically relevant, were entered into a multivariate logistic regression model using backward elimination to identify independent factors associated with cognitive impairment. The dependent variable was the presence or absence of cognitive impairment. As an exploratory complementary analysis, a random forest classification model was constructed using default parameters (ntree = 100) to rank the relative importance of candidate predictors. This analysis was performed solely for variable importance assessment and was not intended as a predictive modeling exercise, and no cross-validation was conducted. All statistical tests were two-sided, and a *P*-value < 0.05 was considered statistically significant.

## 4 Results

### 4.1 Prevalence and univariate comparisons

Among the 220 enrolled older inpatients with chronic heart failure, 123 (55.9%) were identified as having cognitive impairment. The prevalence of cognitive impairment increased significantly with advancing age, with rates of 27.5%, 40.2%, and 82.8% in the 60–69, 70–79, and ≥80 years age strata, respectively (*P* < 0.001). Conversely, cognitive impairment prevalence declined as educational attainment rose, ranging from 79.3% in those with primary education or less, to 56.8% in those with junior high school education, and 28.3% in those with senior high school education or above (*P* < 0.001). Stratification by left ventricular ejection fraction (LVEF) category revealed marked differences in cognitive impairment prevalence: 82.5% in HFrEF, 59.7% in HFmrEF, and 14.5% in HFpEF (*P* < 0.001). Furthermore, patients with cognitive impairment exhibited significantly higher NT-proBNP levels compared with cognitively intact counterparts (*P* < 0.001). The prevalence of cognitive impairment was also significantly elevated among patients with comorbid diabetes, sleep disturbance, or a history of smoking (*P* < 0.05). Detailed comparisons are provided in Tables 1–3.

**TABLE 1:** Comparison of characteristics between elderly hospitalized patients with chronic heart failure with and without cognitive impairment (*n*, %).

Variable		With cognitive impairment (*n* = 123)	No cognitive impairment (*n* = 97)	X^2^/Z	*P*
Gender	Male	63 (49.50)	48 (50.50)	0.065	0.078
	Female	60 (51.20)	49 (48.80)		
Age	60–69	11 (8.90)	29 (29.90)	49.045	<0.001
	70–79	35 (28.50)	52 (53.60)		
	≥80	77 (62.60)	16 (16.50)		
Marital status	Married	109 (88.60)	79 (81.40)	2.297	0.317
	Unmarried	6 (4.90)	7 (7.20)		
	Widowed or divorced	8 (6.50)	11 (11.30)		
Educational level	Primary school and below	46 (37.40)	12 (12.40)	29.313	<0.001
	Junior high school	62 (50.40)	47 (48.50)		
	Senior high school, technical secondary school and above	15 (12.20)	38 (39.20)		
BMI	Underweight	16 (13.40)	10 (10.30)	1.301	0.729
	Normal	50 (40.70)	39 (40.20)		
	Overweight	42 (34.10)	39 (40.20)		
	Obesity	15 (12.20)	9 (9.30)		
Smoking status	Non-smoking	53 (43.10)	56 (57.70)	4.651	0.031
	Smoking	70 (56.90)	41 (42.30)		
Drinking status	Non-drinking	64 (52.00)	44 (45.40)	0.966	0.326
	Drinking	59 (48.00)	53 (54.60)		
Sleep status	Good sleep	47 (38.20)	57 (58.80)	9.190	0.002
	Sleep disorder	76 (61.80)	40 (41.20)		
Disease course	<5 years	35 (28.50)	15 (15.50)	5.266	0.072
	5–9 years	71 (57.70)	65 (58.80)		
	≥10 years	17 (13.80)	17 (17.50)		
Etiology	Coronary heart disease	71 (57.70)	66 (68.00)	7.561	0.109
	Hypertensive heart disease	25 (20.30)	12 (12.40)		
	Cardiomyopathy	8 (6.50)	1 (1.00)		
	Valvular heart disease	17 (13.80)	17 (17.50)		
	Other etiologies	2 (1.60)	1 (1.00)		

**TABLE 2:** Comparison of characteristics between elderly hospitalized patients with chronic heart failure with and without cognitive impairment (*n*, %).

Variable		Cognitive impairment (*n* = 123)	No cognitive impairment (*n* = 97)	t/X^2^	*P*
CADPI (whether the stenosis rate of all three coronary arteries is <50%)		22 (17.90)	29 (29.90)	0.053	0.817
Yes					
No		20 (16.30)	21 (21.60)		
Not done		75 (61.00)	45 (46.40)		
Unknown (not brought by the outer court)		6 (4.90)	2 (2.10)		
NYHA grade				1.681	0.432
2		49 (39.80)	47 (48.50)		
3		56 (45.60)	37 (38.10)		
4		18 (14.60)	13 (13.40)		
Left ventricular ejection fraction	<40%	71 (57.70)	15 (15.50)	68.294	<0.01
	40%–49%	43 (35.00)	29 (25.90)		
	≥50%	9 (7.30)	53 (54.60)		
History of hypertension	None	45 (36.60)	38 (39.20)	0.155	0.694
	None	78 (63.40)	59 (60.80)		
History of diabetes	None	42 (34.10)	57 (58.80)	13.278	<0.001
	None	81 (65.90)	40 (41.20)		
History of myocardial infarction	None	101 (82.10)	74 (76.30)	1.131	0.288
	None	22 (17.90)	23 (23.70)		
History of cerebral infarction	None	85 (69.10)	58 (59.80)	2.067	0.151
	None	38 (30.90)	39 (40.20)		

**TABLE 3:** Analysis of clinical and biochemical indicators in elderly patients with chronic heart failure: cognitive impairment group vs. non-cognitive impairment group.

Variable		Cognitive impairment (*n* = 123)	No cognitive impairment (*n* = 97)	Z	*P*
NT-proBNP (pg/ml)				23.393	<0.001
	≤1000	37 (30.10)	60 (61.90)		
	1001–4000	61 (49.60)	30 (30.90)		
	>4000	25 (20.30)	7 (7.20)		
Low-density lipoprotein		3.28 (1.70–4.15)	2.63 (0.96–4.08)	−1.784	0.074
Triglycerides		1.17 (0.75–1.96)	1.16 (0.70–1.72)	−0.720	0.472
Total cholesterol		4.00 (3.07–5.21)	3.62 (2.97–4.71)	−1.014	0.310
Uric acid		387 (296–487)	404 (287–501)	−0.379	0.705
Homocysteine		17.60 (13.17–22.40)	17.50 (13.37–25.23)	−0.485	0.627

This variable was analyzed as a categorical variable derived from a continuous measure; between-group comparisons were performed using the chi-square (χ^2^) test, with statistical significance set at *P* < 0.05.

### 4.2 Univariate analysis of cognitive impairment in CHF

The study subjects were divided into a cognitive impairment group and a non-cognitive impairment group based on the occurrence of cognitive impairment. Univariate analysis was conducted on demographic characteristics, lifestyle, and clinical indicators of the two groups. The results showed that the proportions of patients aged ≥ 80, with an education level of primary school or below, with a history of smoking, with concomitant sleep disorders, with left ventricular ejection fraction < 40%, and with a history of diabetes were significantly higher in the cognitive impairment group than in the non-cognitive impairment group, with statistically significant differences (*P* < 0.05). There were no statistically significant differences between the two groups in terms of gender, marital status, body mass index, alcohol consumption, duration of heart failure, etiology, degree of coronary artery stenosis, NYHA cardiac function classification, and history of hypertension, myocardial infarction, or cerebral infarction (*P* > 0.05). The above results are shown in Tables 1, 2.

The comparison of clinical biochemical parameters between the two groups is presented in Table 3. The distribution of NT-proBNP levels differed significantly between groups (*P* < 0.001), with markedly higher levels observed in the cognitive impairment group relative to the cognitively intact group. In contrast, no statistically significant differences were detected between the two groups for low-density lipoprotein cholesterol, triglycerides, total cholesterol, uric acid, or homocysteine (*P* > 0.05).

### 4.3 Multivariate analysis of cognitive impairment in CHF patients

Variables exhibiting statistical significance in univariate analyses were entered into a multivariate Logistic regression model. Prior to modeling, multicollinearity was assessed using variance inflation factors (VIF), with all VIF values < 5 indicating no significant collinearity. Multivariate analysis identified the following independent risk factors for cognitive impairment: age ≥ 80 years (OR = 6.122, 95% CI: 2.695–27.979, *P* < 0.001), sleep disturbance (OR = 2.870, 95% CI:1.214–6.789, *P* = 0.016), diabetes mellitus (OR = 2.409, 95% CI: 1.028–5.641, *P* = 0.040), and NT-proBNP > 1000 pg/mL (OR = 3.215, 95% CI: 1.452–7.108, *P* = 0.004). Conversely, educational attainment of senior high school or above (OR = 0.186, 95% CI: 0.055–0.628, *P* = 0.007) and LVEF ≥ 50% (OR = 0.035, 95% CI: 0.012–0.102, *P* < 0.001) emerged as independent protective factors. Variable assignments are presented in Table 4, multicollinearity diagnostics in Table 5, and multivariate logistic regression results in Table 6.

**TABLE 4:** Assignment status.

Variable name assignment	Assignment
Age	Age 60–69 years = 0, 70–79 years = 1, ≥80 years = 2
Education level	Primary school and below = 0, Junior high school = 1, High school, technical school and above = 2
Ejection fraction	<0.4 = 0, 0.4–0.49 = 1, ≥0.5 = 2
Smoking	No = 0, Yes = 1
Diabetes	No = 0, Yes = 1
Sleep	Good sleep = 0, Sleep disorder = 1
NT-proBNP	<1000 = 0, 1000–4000 = 1, >4000 = 2

**TABLE 5:** Results of multicollinearity test for variables in multivariate logistic regression on factors affecting cognitive impairment in elderly patients hospitalized with chronic heart failure.

Variable	Tolerance	VIF	Condition index	Eigenvalue
Constant	0.908	1.102	1.000	7.235
NT-proBNP			2156	1542
Diabetes	0.954	1.048	2318	1327
LVEF	0.843	1.186	2574	1089
Education level	0.892	1.121	2247	1431
Sleep	0.961	1.040	2189	1498
Smoking	0.900	1.112	2213	1465
Age	0.829	1.206	2631	1034

A VIF value greater than 5 indicates moderate multicollinearity, and greater than 10 indicates severe multicollinearity. All VIF values in this table are well below 5.

**TABLE 6:** Multivariate logistic regression analysis results of factors affecting cognitive impairment in elderly patients hospitalized with chronic heart failure.

Variable		β	SE	WaldX^2^	*P*	OR	95% CI
Age							
	60–69	0.291	0.565	0.265	0.607	1.000	0.442–4.052 2.695–27.979
	69–79					1.338	
	≥80	2.210	0.622	12.635	<0.001	6.122	
Education level							
	Primary school and below	0.835	0.549	2.313	0.128	1.000	0.148–1.273 0.055–0.626
	Middle school					0.434	
	High school, technical secondary school and above	1.684	0.621	7.367	0.007	0.186	
LVEF							
	≤0.4	−0.361	0.506	0.508	0.476	1.000	0.259–1.880 0.011–0.116
	0.40–0.49					0.697	
	≥0.5	−3.351	0.612	29.948	<0.001	0.035	
Diabetes							
	No	0.879	0.429	4.207	0.040	1.000	1.040–5.580
	Yes					2.409	
Sleep condition							
	Good sleep	1.054	0.436	5.858	0.016	1.000	1.222–6.741
	Sleep disorders					2.870	
NT-proBNP (pg/ml)							
	≤1000	1.633	0.493	10.982	<0.001	1.000	1.949–13.452 1.673–27.306
	1001–4000					5.120	
	>4000	1.911	0.712	7.193	0.007	6.758	
常量		0.919	0.802	1.312	0.252	0.399	

OR, odds ratio; CI, confidence interval; LVEF, left ventricular ejection fraction. LVEF categories were defined as follows: ≤40% (heart failure with reduced ejection fraction, HFrEF), 40%–49% (heart failure with mildly reduced ejection fraction, HFmrEF), and ≥50% (heart failure with preserved ejection fraction, HFpEF).

### 4.4 Variable importance assessment for cognitive impairment

**FIGURE 1:**
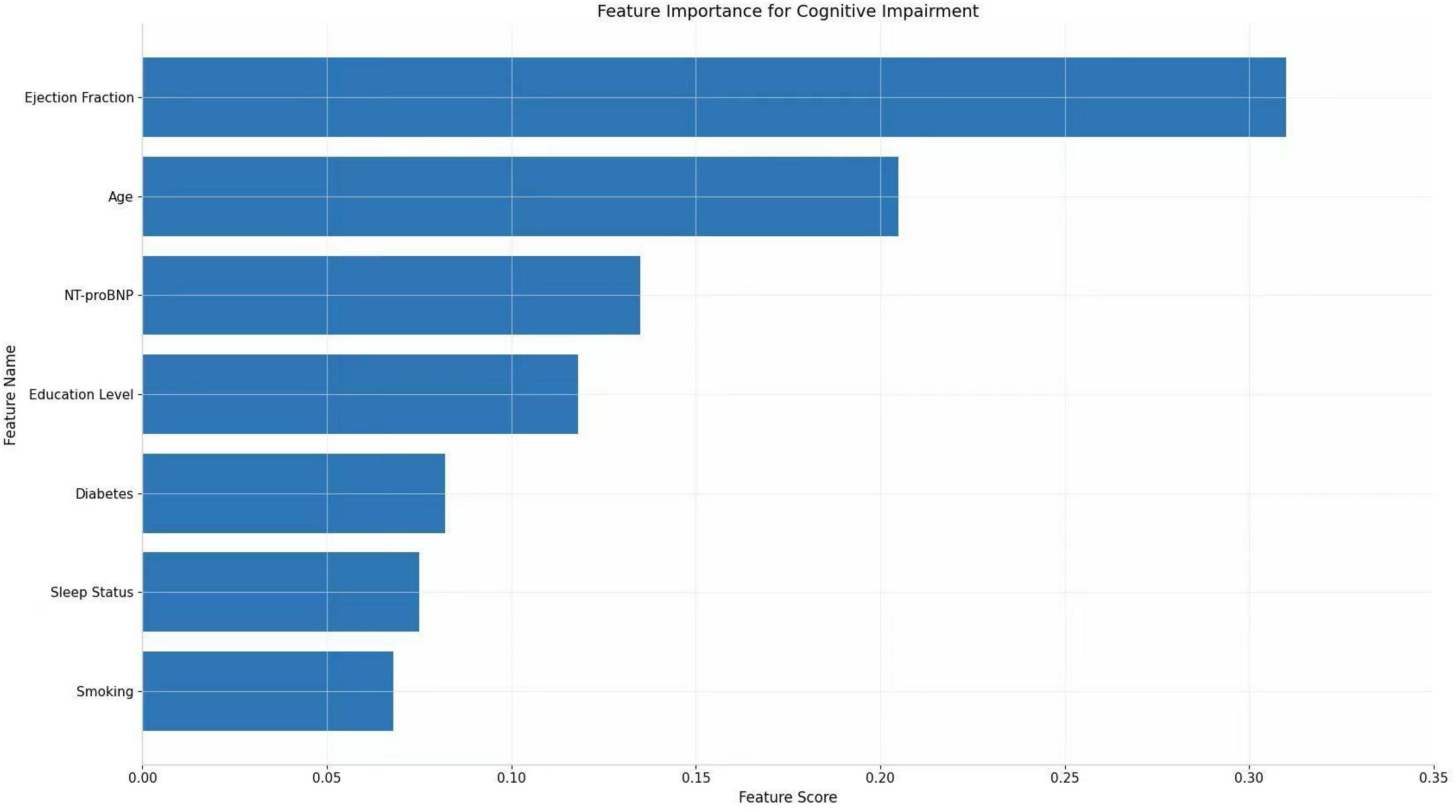
Importance analysis of factors for cognitive impairment in elderly inpatients with chronic heart failure using random forest.

To further elucidate the relative contribution of individual factors to cognitive impairment, variable importance was assessed using a random forest model, as illustrated in Figure 1. Left ventricular ejection fraction exhibited the highest importance score (approximately 0.30), indicating its predominant contribution to model discrimination within the study cohort. Age (score ≈0.21) and NT-proBNP level (score ≈0.14) ranked second and third, respectively, underscoring the relevance of these three parameters in the occurrence of cognitive impairment among older inpatients with chronic heart failure. The importance scores for educational attainment, diabetes mellitus, sleep disturbance, and smoking history decreased sequentially, with smoking demonstrating the weakest association (score ≈0.06). These findings corroborate the results of the multivariate logistic regression analysis and further highlight the pivotal roles of left ventricular ejection fraction, age, and related factors in the development of cognitive impairment.

## 5 Discussion

### 5.1 Analysis of the prevalence of CI and CHF

The frequent co-occurrence of cognitive impairment and cardiovascular disease in aging populations is not coincidental–approximately one-quarter of dementia cases are attributable to cardiovascular risk factors (16). Cognitive impairment (CI) is highly prevalent following heart failure, with reported estimates ranging from 31% to 85%, and individuals aged ≥ 65 years with heart failure exhibit a twofold increased risk of CI compared with those without heart failure (13). In the present study, the prevalence of CI among older inpatients with chronic heart failure was 55.9%, falling within the previously reported range. Given the single-center design and limited sample size, this estimate remains preliminary and may not reflect the broader population characteristics. The high burden of CI in heart failure has been identified as a key contributor to increased clinical care complexity (17), and represents an independent risk factor for mortality in these patients (18).

### 5.2 Analysis of factors associated with cognitive impairment in elderly patients hospitalized with chronic heart failure

#### 5.2.1 Age

The present study identified age ≥ 80 years as an independent risk factor for cognitive impairment in older inpatients with chronic heart failure, with an approximately sixfold increased risk compared with those aged 60–69 years (OR = 6.122, 95% CI: 2.695–27.979, *P* < 0.01), demonstrating a clear age-dependent cumulative effect consistent with prior evidence (19, 20). Although the risk was slightly elevated in the 70–79 years age group (OR = 1.338), the difference was not statistically significant (*P* = 0.607), which may reflect greater cognitive reserve, limited sample size, or incomplete control of confounding factors. A study of 1846 participants similarly confirmed that advanced age, lower educational attainment, and female sex are high-risk factors for cognitive impairment in heart failure (21). Aging exacerbates cognitive decline through multiple synergistic mechanisms with cardiac dysfunction: impaired cerebrovascular regulation increases the risk of cerebral hypoperfusion in critical brain regions when LVEF falls below 40% (22); cerebral β-amyloid deposition and chronic hypoxia exert synergistic neurotoxicity (23); and comorbid hypertension and diabetes further elevate cerebral microvascular resistance (24, 25). The impact of age on cognition exhibits a “dynamic threshold” characteristic–deteriorating cardiac function and metabolic comorbidities jointly reduce cerebral perfusion reserve, accelerating cognitive decline.

Given that age ≥ 80 years is an independent risk factor, this subgroup shows a particularly high risk of cognitive impairment and deserves increased clinical awareness.

### 5.2.2 Education level

In the cognitive impairment group, 87.80% of patients had an educational attainment of junior high school or below, whereas only 12.20% had completed senior high school or above. Multivariate analysis identified educational attainment of senior high school or above as an independent protective factor against cognitive impairment in older inpatients with chronic heart failure (OR = 0.186, 95% CI: 0.055–0.626, *P* = 0.007), with risk reduced to 0.186-fold that of the primary education or below group, demonstrating a dose–response relationship. This finding aligns with existing evidence that higher education confers protection through enhanced cognitive reserve (26). Education, as a cornerstone of cognitive reserve, promotes neuroplasticity and neural efficiency, facilitating adaptive compensation against underlying brain pathology (27); individuals with greater educational attainment possess superior synaptic reserve, enabling sustained cognitive function despite age-related decline (28). Conversely, lower education is associated with diminished cognitive reserve and limited compensatory capacity against neurodegeneration (29).

Among patients with heart failure, higher education (≥12 years) confers dual protection: it optimizes cardiometabolic status, thereby lowering baseline cognitive impairment risk (30); and enhances neural network plasticity–evidenced by increased prefrontal cortical activation during cognitive tasks–to buffer cognitive decline (31). In contrast, lower educational attainment is frequently accompanied by inadequate health literacy and reduced healthcare access, exacerbating cognitive load and negative mood states that further impair cognition (32). These findings underscore the clinical imperative to identify heart failure patients with lower educational attainment as a high-risk subgroup warranting prioritized cognitive screening and provide a theoretical foundation for targeted interventions such as working memory training.

#### 5.2.3 Sleep disorders

The present study identified sleep disturbance as a significant independent risk factor for cognitive impairment in patients with chronic heart failure, with affected individuals exhibiting a 2.870-fold increased risk compared with those reporting good sleep quality. Prior research has likewise demonstrated a positive correlation between cognitive function and overall sleep quality (*r* = 0.322, *P* < 0.01) (33). A bidirectional relationship is postulated: chronic poor sleep may precipitate cognitive decline via impaired memory consolidation and heightened neuroinflammation (34) whereas cognitive impairment itself may disrupt sleep–wake regulation and provoke anxiety, further compromising sleep (35) negative affective states such as anxiety and depression may mediate this association (36). Elevated serum inflammatory markers (e.g., IL-6, CRP) have been documented in heart failure patients with comorbid sleep disturbance (37). As confounding factors (e.g., advanced age) have not been fully controlled, the independent contribution of sleep disturbance warrants further investigation. The close association between sleep disturbance and cognitive impairment suggests that sleep status deserves attention in the clinical evaluation of elderly patients with chronic heart failure.

#### 5.2.4 Diabetes

The present study identified diabetes mellitus as an independent risk factor for cognitive impairment in older inpatients with chronic heart failure (OR = 2.409, 95% CI: 1.040–5.580, *P* = 0.040), conferring a 2.409-fold increased risk compared with non-diabetic patients, consistent with prior evidence (38). Type 2 diabetes mellitus is particularly prevalent among heart failure patients with comorbid cognitive impairment and is associated with elevated mortality and diminished quality of life (1).

Diabetes exacerbates cognitive decline through multiple pathways: hyperglycemia and insulin resistance impair vascular endothelial function, reduce nitric oxide bioavailability, and upregulate protein kinase C expression, thereby accelerating heart failure progression (39); accumulation of advanced glycation end-products inflicts sustained neurovascular injury; and microvascular complications further compromise cerebral perfusion. These mechanisms perpetuate a vicious cycle of “glycemic dysregulation → worsening heart failure → cognitive deterioration” (40). Community-based studies corroborate that individuals with impaired glycemic status exhibit significantly lower MMSE scores than normoglycemic counterparts (41). Clinical evidence supports that metformin and consistent antihyperglycemic therapy may attenuate cognitive decline and reduce dementia risk (42, 43). The close association between diabetes and cognitive impairment observed in this study is consistent with the impact of chronic metabolic disorders on cerebrovascular health and cognitive function.

#### 5.2.5 NT-proBNP

The present study demonstrated a significant positive association between NT-proBNP levels and cognitive impairment risk, with a graded increase in risk across strata of ≤1000, 1001–4000, and >4000 pg/mL (*P* < 0.001 and *P* = 0.007), corroborating the established role of NT-proBNP as a core biomarker of heart failure severity (44).

N-terminal pro-B-type natriuretic peptide is endorsed by European guidelines as a key diagnostic indicator for heart failure (45) and emerging evidence has linked elevated NT-proBNP to an increased risk of cognitive impairment and incident dementia (46). Pathophysiological mechanisms involve two interrelated pathways: elevated NT-proBNP signifies increased ventricular wall stress and reduced cardiac output, culminating in cerebral hypoperfusion and ischemic-hypoxic injury to critical brain regions (47). Concurrently, it reflects overactivation of the renin–angiotensin–aldosterone system and sympathetic nervous system, provoking cerebral vasoconstriction and neurotransmitter dysregulation that accelerate cognitive decline. Longitudinal studies support the dynamic predictive utility of NT-proBNP for cognitive deterioration (48) with both the Rotterdam Study and the Irish Longitudinal Study on Aging identifying it as an independent risk factor for cognitive decline (49, 50). Collectively, NT-proBNP may serve as a potential biomarker for identifying elderly heart failure patients at higher risk of concurrent cognitive impairment.

#### 5.2.6 Left ventricular ejection fraction

Multivariate analysis revealed that patients with LVEF ≥ 50% had a 96.5% lower risk of cognitive impairment compared with those with LVEF ≤ 40% (OR = 0.035, *P* < 0.001). However, the large effect size and wide confidence interval likely reflect the limited number of cognitively impaired cases in the HFpEF subgroup (*n* = 9) and unbalanced group sizes, which constrain the precision of the estimate. Although a trend toward reduced risk was observed in the LVEF 41%–49% group, the difference was not statistically significant, underscoring the need for continued vigilance in clinical monitoring. Prior studies have likewise documented associations between systolic heart failure and cognitive impairment (51, 52), with elevated cognitive impairment risk observed across both HFrEF and HFpEF phenotypes compared with non-HF populations (53).

Declining LVEF exacerbates cognitive decline through multiple pathways: reduced cardiac output precipitates cerebral hypoperfusion, ischemic-hippocampal and prefrontal injury, impaired clearance of metabolic waste, and accelerated Aβ deposition and white matter pathology (54) LRAAS and sympathetic overactivation disrupts cerebrovascular endothelial integrity and neurotransmitter homeostasis (55) systemic hypoperfusion promotes release of proinflammatory cytokines such as TNF-α and IL-6, perpetuating a vicious cycle of cardiac dysfunction, neuroinflammation, and cerebral injury (51). Shang et al. (56) demonstrated that reduced LVEF serves as a key mediator of the association between coronary artery disease and cognitive impairment, with an inverse correlation persisting after adjustment (OR = 0.928, *P* = 0.004). Accordingly, reduced LVEF is closely related to cognitive impairment in elderly heart failure patients, suggesting that cardiac function status is closely linked to cognitive status.

## 6 Conclusion

This study shows that the prevalence of cognitive impairment (CI) in elderly hospitalized patients with chronic heart failure is 55.9%. Age ≥ 80 years, BNP > 4000 pg/ml, coexisting diabetes, and sleep disorders are independent risk factors for CI, while high school education or above and LVEF ≥ 0.5 are independent protective factors (all *P* < 0.05). Smoking has no independent effect, and its impact is influenced by strong confounding factors. These findings clarify the prevalence and related factors of cognitive impairment in elderly hospitalized patients with chronic heart failure, and enhance clinical awareness of cognitive status in this population.

## 7 Limitations

Several limitations should be noted. First, the single-center, cross-sectional design precludes causal inference and may limit the generalizability of our findings. Second, the wide 95% confidence intervals for some effect estimates reflect constraints related to sample size and group imbalance. Third, cognitive status was assessed solely via MMSE screening rather than formal clinical diagnosis. Fourth, residual confounding from unmeasured factors–including nutritional status, medication use, and psychological conditions–cannot be fully excluded. Finally, the assessment of sleep disturbance was not based on a standardized instrument. Future multicenter prospective studies employing validated tools such as the PSQI are warranted to validate these associations and enhance reliability.

## Data Availability

The raw data supporting the conclusions of this article will be made available by the authors, without undue reservation.

## References

[1] Ehtewish H Arredouani A El-Agnaf O. Diagnostic, prognostic, and mechanistic biomarkers of diabetes mellitus-associated cognitive decline. *Int J Mol Sci.* (2022) 23:6144. 10.3390/ijms23116144 35682821 PMC9181591

[2] Wang H Chai K Du M Wang S Cai J Li Y et al. Prevalence and incidence of heart failure among Urban patients in China: a national population-based analysis. *Circ Heart Fail.* (2021) 14:e008406. 10.1161/CIRCHEARTFAILURE.121.008406 34455858

[3] Hao G Wang X Chen Z Zhang L Zhang Y Wei B et al. Prevalence of heart failure and left ventricular dysfunction in China: the China hypertension survey, 2012-2015. *Eur J Heart Fail.* (2019) 21:1329–37. 10.1002/ejhf.1629 31746111

[4] Hugo J Ganguli M. Dementia and cognitive impairment: epidemiology, diagnosis, and treatment. *Clin Geriatr Med.* (2014) 30:421–42. 10.1016/j.cger.2014.04.001 25037289 PMC4104432

[5] Petersen RC Lopez O Armstrong MJ Getchius T Ganguli M Gloss D et al. Practice guideline update summary: mild cognitive impairment [RETIRED]: report of the guideline development, dissemination, and implementation subcommittee of the American academy of neurology. *Neurology.* (2018) 90:126–35. 10.1212/WNL.0000000000004826 29282327 PMC5772157

[6] Jung M Apostolova L Gao S Burney H Lai D Saykin A et al. Association of heart failure with cognitive decline and development of mild cognitive impairment and dementia. *J Cardiovasc Nurs.* (2024) 39:E80–5. 10.1097/JCN.0000000000001075 39137265 PMC11322628

[7] Dong Y Teo S Kang K Tan M Ling L Yeo P et al. Cognitive impairment in Asian patients with heart failure: prevalence, biomarkers, clinical correlates, and outcomes. *Eur J Heart Fail.* (2019) 21:688–90. 10.1002/ejhf.1442 30938010

[8] Murad K Goff D Morgan T Burke G Bartz T Kizer J et al. Burden of comorbidities and functional and cognitive impairments in elderly patients at the initial diagnosis of heart failure and their impact on total mortality: the cardiovascular health study. *JACC Heart Fail.* (2015) 3:542–50. 10.1016/j.jchf.2015.03.004 26160370 PMC4499113

[9] Kim J Hwang S Kim S Shim J. Structural relationships between cognitive function, depressive symptoms, self-care confidence, and maintenance in patients with heart failure. *SAGE Open Nurs.* (2023) 9:23779608231196665. 10.1177/23779608231196665 37691722 PMC10483967

[10] Ovsenik A Podbregar M Fabjan A. Cerebral blood flow impairment and cognitive decline in heart failure. *Brain Behav.* (2021) 11:e02176. 10.1002/brb3.2176 33991075 PMC8213942

[11] Arslan A Çelik A Döven O. The role of biomarkers in predicting cognitive impairment in elderly patients with heart failure. *Turk Kardiyol Dern Ars.* (2024) 52:244–52. 10.5543/tkda.2024.97143 38829644

[12] Peduzzi P Concato J Kemper E Holford TR Feinstein AR. A simulation study of the number of events per variable in logistic regression analysis. *J Clin Epidemiol.* (1996) 49:1373–9. 10.1016/s0895-4356(96)00236-3 8970487

[13] Yohannes A Chen W Moga A Leroi I Connolly M. Cognitive impairment in chronic obstructive pulmonary disease and chronic heart failure: a systematic review and meta-analysis of observational studies. *J Am Med Dir Assoc.* (2017) 18:451.e1–11. 10.1016/j.jamda.2017.01.014 28292570

[14] Januzzi J van Kimmenade R Lainchbury J Bayes-Genis A Ordonez-Llanos J Santalo-Bel M et al. NT-proBNP testing for diagnosis and short-term prognosis in acute destabilized heart failure: an international pooled analysis of 1256 patients: the international collaborative of NT-proBNP study. *Eur Heart J.* (2006) 27:330–7. 10.1093/eurheartj/ehi631 16293638

[15] Zhang MY. Selection and comparison of rating scales. *Shanghai Arch Psychiatry*. (1991) 75–82.

[16] Tsao C Aday A Almarzooq Z Alonso A Beaton A Bittencourt M et al. Heart disease and stroke statistics-2022 update: a report from the American heart association. *Circulation.* (2022) 145:e153–639. 10.1161/CIR.0000000000001052 35078371

[17] Wleklik M Diakowska D Lee C Vellone E Lisiak M Szczepanowski R et al. Risk factors for adverse clinical outcomes in elderly patients with heart failure and cognitive frailty. *Appl Nurs Res.* (2026) 87:152043. 10.1016/j.apnr.2025.152043 41578990

[18] Álvarez-García J. Cognitive dysfunction in heart failure with preserved ejection fraction: uncovering the consequences of an overlooked comorbidity. *Circulation.* (2024) 150:1928–30. 10.1161/CIRCULATIONAHA.124.072220 39652649

[19] Bolaji O Bahar Y Lohana S Bahar A Lawrence I Mazimba S. The heart-brain axis: neurocognitive frailty in heart failure. *J Neurol.* (2025) 272:522. 10.1007/s00415-025-13257-z 40690005

[20] He H Wei M Zhong J Wang J Huang L Lan Y et al. Biological aging affects the rate of cognitive decline in middle-aged and elderly populations: a cohort study based on CHARLS. *Sichuan da xue xue bao Yi xue ban.* (2025) 56:470–7. 10.12182/20250360203 40599287 PMC12207056

[21] Lee T Qian M Liu Y Graham S Mann D Nakanishi K et al. Cognitive decline over time in patients with systolic heart failure: insights from WARCEF. *JACC Heart Fail.* (2019) 7:1042–53. 10.1016/j.jchf.2019.09.003 31779926 PMC6944056

[22] Hillier E Covone J Fischer K Chen H Hafyane T Friedrich M. Microvascular dysfunction as a possible link between heart failure and cognitive dysfunction. *Circ Heart Fail.* (2023) 16:e010117. 10.1161/CIRCHEARTFAILURE.122.010117 37750336

[23] Troncone L Luciani M Coggins M Wilker E Ho C Codispoti K et al. Aβ Amyloid pathology affects the hearts of patients With Alzheimer’s disease: mind the heart. *J Am Coll Cardiol.* (2016) 68:2395–407. 10.1016/j.jacc.2016.08.073 27908343 PMC5142757

[24] Feuer D Handberg E Mehrad B Wei J Bairey Merz C Pepine C et al. Microvascular dysfunction as a systemic disease: a review of the evidence. *Am J Med.* (2022) 135:1059–68. 10.1016/j.amjmed.2022.04.006 35472396 PMC9427712

[25] Katsi V Mavroudis A Liatakis I Konstantinos M Tsioufis K. Exploring the relationship between hypertension and cerebral microvascular disease. *Diseases.* (2024) 12:266. 10.3390/diseases12110266 39589940 PMC11592893

[26] Klee M Aho V May P Heintz-Buschart A Landoulsi Z Jónsdóttir S et al. Education as risk factor of mild cognitive impairment: the link to the gut microbiome. *J Prev Alzheimers Dis.* (2024) 11:759–68. 10.14283/jpad.2024.19 38706292 PMC11060993

[27] Cipriani GE Molfese S Giovannelli F Güntekin B Vitali N Marcato R et al. Executive control from healthy ageing to cognitive impairment: a systematic review of stroop and simon effects using psychophysiological and imaging techniques. *Neurosci Biobehav Rev.* (2025) 172:106121. 10.1016/j.neubiorev.2025.106121 40139290

[28] Kwan E Draper B Endre Z Harvey S Brown M. Prevalence, types and recognition of cognitive impairment in dialysis patients in South Eastern sydney. *Intern Med J.* (2021) 51:2034–41. 10.1111/imj.14976 32672898

[29] Han X Li Y Wang J Liu X Zhang Y Dong Q et al. Associations between lifelong cognitive reserve, Alzheimer’s disease-related plasma biomarkers, and cognitive function in dementia-free older adults: a population-based study. *J Alzheimers Dis.* (2025) 103:821–32. 10.1177/13872877241306448 39791378

[30] Bleil M Roisman G Hamilton D Magro S Appelhans B Gregorich S et al. Which aspects of education are health protective? a life course examination of early education and adulthood cardiometabolic health in the 30-year study of early child care and youth development (SECCYD). *BMC Public Health.* (2024) 24:1092. 10.1186/s12889-024-18560-4 38641792 PMC11031877

[31] Coors A Lee S Gazes Y Gacheru M Habeck C Stern Y. Brain reserve affects the expression of cognitive reserve networks. *Hum Brain Mapp.* (2024) 45:e26658. 10.1002/hbm.26658 38520368 PMC10960550

[32] Han S Boyle P James B Yu L Bennett D. Poorer financial and health literacy among community-dwelling older adults with mild cognitive impairment. *J Aging Health.* (2015) 27:1105–17. 10.1177/0898264315577780 25903976 PMC4520748

[33] Gan Y Liao T Du Y Liu L Luo L Huang W et al. Association between mild cognitive impairment and sleep quality in patients with chronic heart failure: a cross-sectional study. *Front Psychiatry.* (2025) 16:1704672. 10.3389/fpsyt.2025.1704672 41293200 PMC12641006

[34] Yuan X Wang C. Sleep deprivation-induced cognitive impairment: unraveling the role of neuroinflammation. *Exp Neurol.* (2025) 394:115419. 10.1016/j.expneurol.2025.115419 40818666

[35] Gilstrap L Gorodeski E Goyal P. Heart failure and cognitive impairment: complexity that requires a new approach. *J Am Geriatr Soc.* (2022) 70:1652–4. 10.1111/jgs.17779 35426956 PMC9177752

[36] Mo W Liu X Yamakawa M. Prevalence of sleep disturbances in people with mild cognitive impairment: a systematic review protocol. *JBI Evid Synth.* (2023) 21:2211–7. 10.11124/JBIES-22-00438 37338281

[37] Imani M Sadeghi M Khazaie H Emami M Sadeghi Bahmani D Brand S. Evaluation of serum and plasma interleukin-6 levels in obstructive sleep apnea syndrome: a meta-analysis and meta-regression. *Front Immunol.* (2020) 11:1343. 10.3389/fimmu.2020.01343 32793188 PMC7385225

[38] Damanik J Yunir E. Type 2 diabetes mellitus and cognitive impairment. *Acta Med Indonesiana.* (2021) 53:213–20. 34251351

[39] Sangha G Smith L Kheradmand M Munir K Rangachar N Weber C et al. Piezo1 activates nitric oxide synthase in red blood cells via protein kinase C with increased activity in diabetes. *Mechanobiol Med.* (2025) 3:100145. 10.1016/j.mbm.2025.100145 40809083 PMC12344281

[40] Liu Y Meng R Dong J. Effect of chronic heart failure complicated with type 2 diabetes mellitus on cognitive function in the elderly. *Evid Based Complement Alternat Med.* (2022) 2022:4841205. 10.1155/2022/4841205 35800008 PMC9256388

[41] Xiu S Zheng Z Liao Q Chan P. Different risk factors for cognitive impairment among community-dwelling elderly, with impaired fasting glucose or diabetes. *Diabetes Metab Syndrome Obesity Targets Therapy.* (2019) 12:121–30. 10.2147/DMSO.S180781 30666140 PMC6330976

[42] Li Z Li S Xiao Y Zhong T Yu X Wang L. Nutritional intervention for diabetes mellitus with Alzheimer’s disease. *Front Nutr.* (2022) 9:1046726. 10.3389/fnut.2022.1046726 36458172 PMC9707640

[43] Andrews S Fulton-Howard B O’Reilly P Marcora E Goate A. Causal associations between modifiable risk factors and the Alzheimer’s phenome. *Ann Neurol.* (2021) 89:54–65. 10.1002/ana.25918 32996171 PMC8088901

[44] Cao Z Jia Y Zhu B. BNP and NT-proBNP as diagnostic biomarkers for cardiac dysfunction in both clinical and forensic medicine. *Int J Mol Sci.* (2019) 20:1820. 10.3390/ijms20081820 31013779 PMC6515513

[45] McDonagh TA Metra M Adamo M Gardner RS Baumbach A Bohm M et al. 2023 Focused Update of the 2021 ESC Guidelines for the diagnosis and treatment of acute and chronic heart failure: developed by the task force for the diagnosis and treatment of acute and chronic heart failure of the European society of cardiology (ESC) With the special contribution of the Heart failure association (HFA) of the ESC. *Eur J Heart Fail.* (2024) 26:5–17. 10.1002/ejhf.3024 38169072

[46] Nagata T Ohara T Hata J Sakata S Furuta Y Yoshida D et al. NT-proBNP and risk of dementia in a general japanese elderly population: the hisayama study. *J Am Heart Assoc.* (2019) 8:e011652. 10.1161/JAHA.118.011652 31446828 PMC6755853

[47] Gyanwali B Lai M Lui B Liew O Venketasubramanian N Richards A et al. Blood-based cardiac biomarkers and the risk of cognitive decline, cerebrovascular disease, and clinical events. *Stroke.* (2021) 52:2275–83. 10.1161/STROKEAHA.120.032571 33971742

[48] Ostovaneh M Moazzami K Yoneyama K Venkatesh B Heckbert SR Wu CO et al. Change in NT-proBNP (N-Terminal Pro-B-Type natriuretic peptide) level and risk of dementia in Multi-ethnic study of atherosclerosis (MESA). *Hypertension.* (2020) 75:316–23. 10.1161/HYPERTENSIONAHA.119.13952 31865797 PMC7482429

[49] Goh FQ Kong WK Wong RC Chong Y Chew N Yeo T et al. Cognitive impairment in heart failure-a review. *Biology.* (2022) 11:179. 10.3390/biology11020179 35205045 PMC8869585

[50] Xiao T van der Velpen I Niessen W Tilly M Kavousi M Ikram M et al. NT-proBNP and changes in cognition and global brain structure: the rotterdam study. *Eur J Neurol.* (2023) 30:2230–9. 10.1111/ene.15859 37165557

[51] Dehghan A Giugni F Bartholdy K Yang Y Lamberson V Wang W et al. Left ventricular remodeling and diastolic dysfunction predict cognitive decline in older adults. *Alzheimers Dement.* (2026) 22:e71089. 10.1002/alz.71089 41761847 PMC12949455

[52] Morse C Luchkanych A Boyes N Champagne A Kelly M Nelson M et al. Cardiac dysfunction is associated with indices of brain atrophy and cognitive impairment in heart failure with reduced ejection fraction. *J Appl Physiol.* (2025) 138:1024–33. 10.1152/japplphysiol.00840.2024 40111286

[53] Witt L Rotter J Stearns S Gottesman R Kucharska-Newton A Richey Sharrett A et al. Heart Failure and Cognitive Impairment in the Atherosclerosis Risk in Communities (ARIC) Study. *J Gen Intern Med.* (2018) 33:1721–8. 10.1007/s11606-018-4556-x 30030736 PMC6153245

[54] Sabayan B van Buchem M Sigurdsson S Zhang Q Harris T Gudnason V et al. Cardiac hemodynamics are linked with structural and functional features of brain aging: the age, gene/environment susceptibility (AGES)-Reykjavik Study. *J Am Heart Assoc.* (2015) 4:e001294. 10.1161/JAHA.114.001294 25628405 PMC4330056

[55] Rouch L Hoang T Xia F Sidney S Lima J Yaffe K. Twenty-five-year change in cardiac structure and function and midlife cognition: the CARDIA study. *Neurology.* (2022) 98:e1040–9. 10.1212/WNL.0000000000013249 35082172 PMC8967387

[56] Shang S Liu Z Gao J Wang J Lu W Fei Y et al. The relationship between pre-existing coronary heart disease and cognitive impairment is partly explained by reduced left ventricular ejection fraction in the subjects without clinical heart failure: a cross-sectional study. *Front Hum Neurosci.* (2022) 16:835900. 10.3389/fnhum.2022.835900 35634203 PMC9130859

